# A Community-Based Intervention Program to Enhance Family Communication and Family Well-being: The Learning Families Project in Hong Kong

**DOI:** 10.3389/fpubh.2017.00257

**Published:** 2017-09-29

**Authors:** Chen Shen, Alice Wan, Lit Tung Kwok, Sally Pang, Xin Wang, Sunita M. Stewart, Tai Hing Lam, Sophia Siu Chee Chan

**Affiliations:** ^1^Li Ka Shing Faculty of Medicine, School of Public Health, The University of Hong Kong, Hong Kong, Hong Kong; ^2^Christian Family Service Center, Kwun Tong, Hong Kong; ^3^Department of Psychiatry, The University of Texas Southwestern Medical Center at Dallas, Dallas, TX, United States; ^4^Li Ka Shing Faculty of Medicine, School of Nursing, The University of Hong Kong, Hong Kong, Hong Kong

**Keywords:** community-based intervention, community engagement, family, social ecological model, mediation analysis

## Abstract

**Background:**

Family communication is important to maintain family relationships and family well-being. To enhance family communication and family well-being, a community-based “Learning Families Project,” based on the social ecological model was developed in Kwun Tong in Hong Kong, a district with high prevalence of family problems.

**Methods:**

This quasi-experimental study included two nearby government subsidized low-rent housing estates separated by busy main roads, as the intervention [Tsui Ping (South) Estate] and control (Shun Tin Estate) estate. The main intervention was resident training programs, such as talks, day camps, and thematic activities. No program was implemented in the control estate. Participants in the intervention group received assessments before the intervention (T1), immediately after the intervention (T2), and 6 weeks after the intervention (T3). Control group participants were assessed at baseline (March to April 2011) and follow-up (December 2011 to March 2012). Assessments of family communication (time and perceived adequacy) and family well-being (harmony, happiness, and health) at T1 and T3 were obtained in the intervention group to examine within-group changes. In addition, these differences in outcomes in the intervention group were compared with those in the control group to examine the effectiveness of the intervention.

**Results:**

Family communication time and perceived communication adequacy increased significantly in the intervention group (*n* = 515) with a small effect size (Cohen effect *d*: 0.10 and 0.24, respectively). Compared with the control group (*n* = 476), the improvements in family communication time and perceived communication adequacy (Cohen effect *d*: 0.13 and 0.14, respectively), and perceived family harmony and happiness (Cohen effect *d*: 0.12 and 0.12, respectively) were significantly greater in the intervention group, adjusting for age and education, suggesting the intervention was effective in improving family communication and family well-being. Mediation analysis showed that perceived communication adequacy mediated the effects of the intervention on family harmony [β = 0.10, 95% confidence interval (CI) 0.03, 0.18], happiness (β = 0.12, 95% CI 0.04, 0.20), and health (β = 0.10, 95% CI 0.02, 0.17), adjusting for age and education.

**Conclusion:**

This community intervention based on the social ecological model improved family well-being through improving family communication, which could be an effective target to promote family well-being in other communities.

**Trial registration:**

This study was registered under ClinicalTrials.gov, identifier NCT02851667. URL: https://clinicaltrials.gov/ct2/show/NCT02851667?term=02851667&rank=1.

## Introduction

Family well-being, which has been conceptualized as “family-life satisfaction,” “sense of well-being,” and “family function” (1), is associated with outcomes such as hypertension, self-rated health, depression, and self-esteem in family members (2–5). Inter-personal harmony in the family is believed to be crucial for the Chinese who see it as contributing not only to each individual member’s welfare but also to a well-organized and peaceful world (6, 7). According to traditional Confucius ideals, family harmony is the basis for an individual’s happiness (8). Our qualitative studies in Hong Kong have also found that family health, happiness, and harmony (3Hs) are three major themes of family well-being (9, 10). Family health includes physical and mental health of family members, which is strongly related to psychological capital and family unity (9). Family happiness can be enhanced by spending time with family members and building connection with friends and relatives. Family harmony means absence of conflicts and effective communication with family members. Forbearance and spending time with family are important in forming a harmonious family (9).

Family communication is important for maintaining family well-being because it provides the foundations of a symbolic, transactional process of creating and sharing meanings, thoughts, feelings or ideas for family members as well as seeking satisfaction (11–13). High-quality communication in family has been reported as one dimension of family well-being (14). In addition, communication, especially non-confrontational and mutually supportive communication (harmony) is a way to develop and maintain inter-personal relationships within the members in their social networks such as the family (15, 16).

Kwun Tong is a district in Hong Kong with a high prevalence of family problems such as elderly abuse, domestic violence, and child abuse (17, 18). In order to promote family 3Hs or well-being in Kwun Tong, the “Learning Families Project (LFP)” was delivered with a series of community-based intervention programs. The LFP was part of the project entitled “FAMILY: a Jockey Club Initiative for a Harmonious Society” (the FAMILY Project) that included a longitudinal family cohort study (19), other interventional studies (20–23), and social marketing programs (24). The FAMILY project focused on the family as a unit and aimed to identify the sources of family problems, devise appropriate preventive measures, and promote family 3Hs.

The LFP was initiated based on the social ecological model. This social ecological model proposes dynamic interrelations among various personal and environmental factors (25). The model emphasizes people’s behaviors are affected by intra-personal, inter-personal, community, and societal factors (26). These programs promoted the concepts of Learning Family and family 3Hs to the participants (intra- and inter-personal level), and was innovative in extending across multiple levels of factors. The concepts of Learning Family indicated that family relationship could be improved when family members participate in learning activities together. These programs also provided a platform for family members to learn together and communicate with each other (inter-personal level), as well as for residents to interact in these community activities (community level).

**Figure 1:**
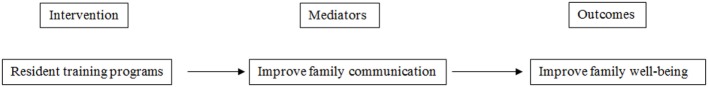
Pathway of intervention on outcomes.

Previous intervention programs using the social ecological model have required intensive involvement from both the service providers and recipients (27, 28), perhaps making such kind of programs difficult to sustain and disseminate. Our previous brief community-based intervention programs, often with a core session and booster session, yielded some small but significant improvement on family communication, parent–child relationships, and family well-being (29, 30). In addition, previous studies on family communication mainly focused on communication patterns, rather than communication time or adequacy (31–33). Our previous telephone survey in Hong Kong showed face-to-face and phone use for family communication associated with a higher level of perceived family well-being (34). This study aimed to examine whether our brief community-based intervention programs could improve family communication and family well-being. We also examined the contribution of family communication by mediation analysis (35), including family communication time and perceived communication adequacy to the improvement in family well-being. The pathway of the intervention on outcomes is shown in Figure 1.

## Materials and Methods

### Participants

Participants were recruited by a large charitable non-governmental social welfare organization, Christian Family Service Center (CFSC), with the mission to support and enhance family functioning and to foster an environment for growth and change in this district. CFSC provides an array of services such as children and family services, youth services, and elderly care services (36). CFSC initiated the Learning Family approach and collaborated with School of Public Health, The University of Hong Kong (HKU) in planning and implementing LFP.

We chose Tsui Ping (South) Estate as the intervention estate, and Shun Tin Estate as the control estate. As the two estates offer government subsidized, public, and low-rent housing, the residents are of similar socio-economic backgrounds. They are located about 2.6 km apart, and are well-separated by busy main roads, minimizing the likelihood of cross-social relationships and transfer. Residents living in the designated estates were eligible if they were Hong Kong residents, older than 10 years of age, and could communicate in Chinese (Cantonese or Putonghua). The headquarter of CFSC is located at a few minutes’ walk from Tsui Ping (South) Estate.

Key community stakeholders such as the Estate Management Advisory Committees (EMAC) and the Mutual Aid Committees (MAC) in the intervention estate were actively engaged as key partners in this study, including joining focus group interviews at the needs assessment stage. The needs assessment was conducted to explore their views on how to promote family 3Hs in Kwun Tong families as well as learning needs, resources, and feasibility of this study. A train-the-trainer program was designed and implemented from December 2010 to February 2011 by HKU and CFSC to engage and equip resident leaders from both EMAC and MAC to recruit participants and organize family programs with the Learning Family concepts and leadership skills. Intervention programs included promotion programs, resident training programs, and learning programs. This paper focuses on resident training programs, which were the main intervention of the LFP.

Fieldwork recruitment of residents took place from March to December 2011. A diverse array of recruitment strategies were used concurrently, including posters, leaflets, and banners, a kick-off ceremony, promotion activities, telephone calls, mobile counter, and door-to-door canvassing to raise awareness of the LFP in the community residents. Resident leaders, CFSC project staff (social workers), and HKU academic staff, were actively involved in the recruitment process.

### Intervention Development

Based on the information from the needs assessment, 24 resident training programs such as talks, day camp, and thematic activities were delivered in the intervention estate by CFSC from June to November 2011. Each program included an introduction to the concepts of Learning Family and family 3Hs as well as how to promote family 3Hs through learning and communicating with family, delivered by interactive games and workshops. The workshops included a variety of topics such as cooking and handwork, enabling family members to learn, and communicate with each other. Resident training programs except for the day camp were held in CFSC headquarters with the duration limited to 2 h to enhance recruitment and reduce costs. The day camp was held in a holiday camp and the duration of contents related to learning family in day camps was also limited to 2 h. Participants completed a questionnaire before (T1) and immediately after the intervention (T2). A 26-page booklet (Learning Family Booklet) produced as a tool for training and also used as a record book for the participants to document their participation in the learning activities as well as their learning contents, was distributed to each participant. The number of participants in the resident training programs was 980, with 515 valid questionnaires at T1 and 444 valid questionnaires at T2.

**Figure 2:**
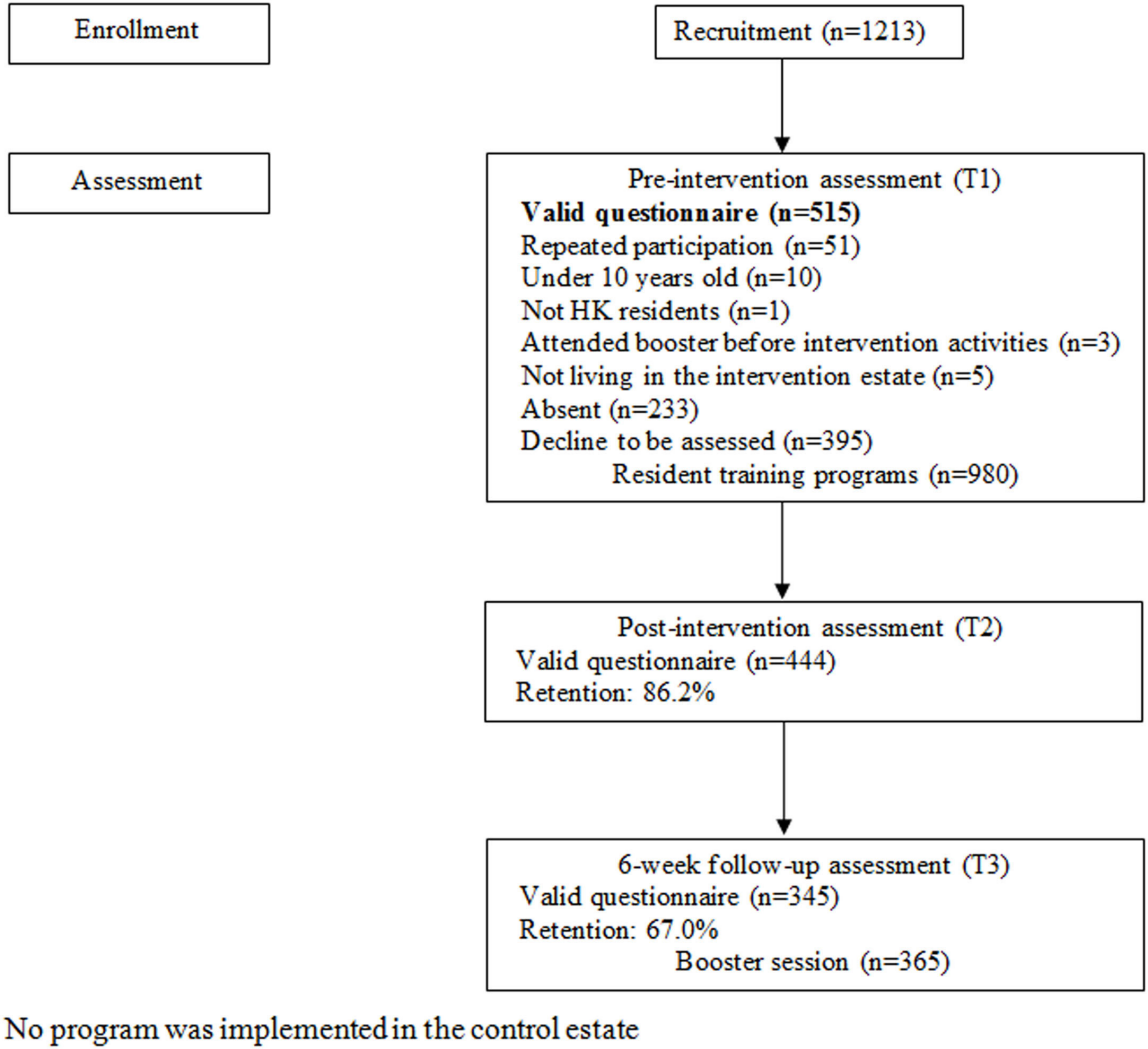
The flow diagram of participants in the intervention group.

A total of six booster sessions were held 6 weeks after the resident training programs for the participants in the New Life Interactive Farm from July to December 2011. The duration of each session was 3 h. Each participant in the booster session had attended one resident training program and had completed a valid questionnaire at T1. The participants had a guided tour of the farm and two experiential activities (organic farming and seed learning). Debriefing and reviews of the concepts of Learning Family and family 3Hs were also provided. Questionnaire assessment was conducted after the booster session (T3). The number of participants in the booster session was 365, with 345 valid questionnaires at T3. Each participant with a valid questionnaire at T3 received one resident training program and one booster session. The flow diagram of the intervention is presented in Figure 2.

### Baseline and Follow-up Surveys

We conducted a baseline (before the intervention programs) and a follow-up survey (after the end of intervention programs) in the intervention and control estates using a self-administrated questionnaire in order to evaluate the whole impact of the programs on the community. The questionnaires were delivered and collected by multiple methods such as mobile counters, security desk in housing blocks, door-to-door visits, and street booths.

A total of 1,167 and 1,108 residents in the intervention and control estates, respectively, participated in the baseline survey, and 1,323 and 1,108 residents in the intervention and control estates, respectively, participated in the follow-up survey. A total of 502 and 476 residents from the intervention and control estate, respectively, were successfully followed up (the same person completed both the baseline and follow-up questionnaires) using record linkage based on the name and residential address. The baseline survey was conducted from March to April 2011, and the follow-up survey was conducted from December 2011 to March 2012.

### Focus Group Interview

Six focus group interviews were conducted with 54 participants who had participated in the resident training programs to explore their experiences in the programs, their mastery of Learning Family concepts, as well as the changes in family communication and family 3Hs. The number of participants in each focus group ranged from 6 to 12.

These interviews were conducted from February to May 2012 in a quiet venue (e.g., an activity room) and lasted about 60 min. Each group was managed by a panel of three members, which consisted of one moderator and two note-takers.

### Outcomes

We used family communication time and perceived communication adequacy as measures of family communication. Participants were asked about their average daily communication time (minutes) with family members in the last 7 days. Perceived communication adequacy was measured by asking the participants whether they felt they had adequate communication with family members. Choices included “not adequate at all,” “not adequate,” “fair,” “adequate,” and “very adequate.” It was measured at T1 and T3 in the intervention group, as well as at baseline and follow-up in the control group. Family 3Hs were assessed by three separate questions which have been used in our previous study (34). Respondents provided a score from 0 to 10 for their perceptions of the health, happiness, and harmony in their own families. We used the information on perceived family 3Hs measured at T1 and T3 in the intervention group, as well as baseline and follow-up in the control group. Family communication and family well-being were not measured at T2 because these cannot change immediately after the intervention.

### Assessments

Participants in the intervention group who received assessments from T1 to T3 (*n* = 515) and respondents who participated in baseline and follow-up surveys in the control group (*n* = 476) were included in the present data analyses. Outcome changes within the intervention group, and comparisons with those in the control group (between group differences in the changes) were assessed to examine the effectiveness of the intervention on family communication and family well-being.

### Fidelity Check

Fidelity of the interventions was monitored by HKU academic and CFSC staff. It included program quality and program objectives achievement, both of which were rated on a single item for adherence to program content, with a score scale ranging from 0 to 100. Fidelity checks showed that the mean scores of both program quality and program objectives achievement were more than 75, indicating that the interventions were delivered to participants successfully as planned.

### Ethical Statement

Ethical approval was granted by the Institutional Review Board (IRB) of the University of Hong Kong/Hospital Authority Hong Kong West Cluster on November 2010. Written informed consent was obtained from all participants before the start of the programs. For participants younger than 15, written consent was obtained from the next of kin, caretakers, or guardians on their behalf. This study was registered under ClinicalTrials.gov (NCT02851667). The authors confirm that all ongoing and related trials for this intervention are registered.

### Statistical Analyses

Chi-square tests and analysis of variance (ANOVA) were used to compare baseline characteristics and outcomes between participants in the intervention and control groups. Paired *t*-test was used to assess the mean change of family communication and perceived family 3Hs in the intervention group. ANOVA was used to assess the difference of mean change of family communication and perceived family 3Hs between the intervention and control groups. Effect size was measured using Cohen’s *d*. A Cohen’s *d* of 0.20 was described as a small effect, 0.50 as a medium effect, and 0.80 or above as a large effect (37).

To identify the relative contribution of family communication, the total effect of the intervention on family 3Hs was decomposed into the direct effect of the intervention (effect unexplained by family communication) and the indirect effect *via* family communication. We performed mediation analysis (35) to obtain the indirect effect, direct effect, and the total effect, adjusting for potential confounders when appropriate. Whether effects varied by age or sex was assessed from the significance of interaction terms and the heterogeneity of estimates across strata.

Multiple imputation was used to handle missing values, which were predicted based on a flexible additive regression model with predictive mean matching incorporating intervention, potential confounders, and outcomes (38). We imputed any missing values 20 times and results from the 20 imputed datasets were summarized into single estimated beta coefficients (β) with confidence intervals (CIs) and *P*-values adjusted for the missing data uncertainty. All the statistical analyses were conducted using STATA version 13.0.

Qualitative data were independently analyzed by a panel of two researchers independently: one attended the focus group interviews, while the other was absent, an arrangement promoting accuracy as well as objectivity during analysis. The strategy of thematic content analysis was used (39). Each transcript was analyzed sentence by sentence and coded for respondents’ meanings. Initial open coding of the data used differing codes, which were then organized into categories and themes.

## Results

Table 1 shows that participants in the intervention group were older and less educated than those in the control group. Otherwise, sex, monthly household income, and marital status were similar. Communication time and perceived communication adequacy were lower in the intervention group than the control group, but perceived family 3Hs were similar. There were no significant differences in baseline demographic characteristics, mediators or outcomes between those who completed questionnaires at both T1 and T3 and those who were lost to follow-up at T3 (all *P*-values larger than 0.05, data not shown).

**Table 1:** Baseline demographic characteristics, mediators, and outcomes between participants in the intervention and control group.

	Intervention group (*n* = 515)	Control group (*n* = 476)	*P*-values^a^
Sex (%)			
Men	27.5	29.8	
Women	72.5	70.2	0.46
Age (%)			
<18	12.2	3.4	
18–44	26.4	33.9	
45–64	25.0	32.4	
65+	36.5	30.3	<0.001
Education level (%)			
No formal education	20.1	14.0	
Primary	30.9	28.8	
Secondary or above	49.1	57.2	0.01
Monthly household income (%)			
<HK$4000 (US$1 = HK$7.8)	20.7	18.6	
4,000–7,999	22.2	18.8	
8,000–9,999	16.7	18.0	
10,000–14,999	21.5	20.6	
≥15,000	18.9	24.0	0.46
Marital status (%)			
Single	18.2	22.5	
Married or cohabitated	59.0	57.9	
Divorced or widowed	22.8	19.6	0.18

Communication time (min)^b^	108.7 (108.6)	144.5 (139.3)	<0.001
Perceived communication adequacy^b^	3.11 (0.97)	3.47 (0.95)	<0.001
Family harmony^b^	7.50 (2.13)	7.71 (2.00)	0.13
Family happiness^b^	7.29 (2.15)	7.28 (2.11)	0.96
Family health^b^	7.17 (2.10)	7.00 (2.08)	0.22

*^a^P-values for two-sided χ^2^ test for demographic characteristics and analysis of variance for mediators and outcomes*.

*^b^Mean (SD)*.

Table 2 shows that communication time and perceived communication adequacy increased significantly at T3 with a small effect size (Cohen effect *d*: 0.10 and 0.24, respectively). Perceived family 3Hs increased at T3 insignificantly.

**Table 2:** The mean change of family communication and family 3Hs in the intervention group.

	Mean (SD)		Mean change	*P*-values	Effect size^a^
	T1	T3			
Communication time (min)	108.7 (108.6)	122.1 (119.2)	21.2	0.02	**0.10**
Perceived communication adequacy^b^	3.11 (0.97)	3.43 (0.88)	0.33	<0.001	**0.24**
Family harmony^c^	7.50 (2.13)	7.67 (1.68)	0.26	0.06	0.08
Family happiness	7.29 (2.15)	7.43 (1.85)	0.16	0.18	0.06
Family health	7.17 (2.10)	7.32 (1.91)	0.11	0.40	0.04

*T1, baseline; T3, 6-week follow-up*.

*P < 0.05 are marked in bold*.

*^a^Cohen effect size index d: 0.20 = small, 0.50 = medium, 0.80 = large*.

*^b^Scores for perceived communication adequacy ranged from 1 to 5, with higher scores indicating better outcomes*.

*^c^Scores for family harmony, happiness, and health ranged from 0 to 10, with higher scores indicating better outcomes*.

Table 3 shows that the improvements in communication time, perceived communication adequacy, family harmony, and happiness were significantly greater in the intervention group than the control group, with a small effect size (Cohen effect *d*: 0.13, 0.14, 0.12, and 0.12, respectively), after adjusting for age and education. These results suggested effectiveness of the intervention on improving family communication and family well-being.

**Table 3:** Differences in changes of family communication and family 3Hs between the intervention and control group.

	Intervention group	Control group	*F* statistic	*P*-values	Effect size^b^
	Mean change^a^ (T3–T1)	Mean change (follow up-baseline)			
Communication time (min)	21.2	−18.0	3.87	0.01	**0.13**
Perceived communication adequacy	0.33	0.02	5.12	0.002	**0.14**
Family harmony	0.26	−0.04	3.82	0.01	**0.12**
Family happiness	0.16	−0.04	3.79	0.01	**0.12**
Family health	0.11	0.10	1.97	0.12	0.09

*T1, baseline; T3, 6-week follow-up*.

*Adjusted for age and education*.

*P < 0.05 are marked in bold*.

*^a^Positive changes in scores indicated improved outcomes*.

*^b^Cohen effect size index d: 0.20 = small, 0.50 = medium, 0.80 = large*.

Table 4 shows that perceived communication adequacy mediated the effect of intervention on perceived family harmony (β = 0.10, 95% CI 0.03, 0.18), family happiness (β = 0.12, 95% CI 0.04, 0.20), and family health (β = 0.10, 95% CI 0.02, 0.17), adjusted for age and education. However, family communication time showed no mediation effect. All the effects did not vary by age or sex (all *P*-values for interaction larger than 0.05).

**Table 4:** Adjusted^a^ indirect, direct, and total effect of intervention on family 3Hs mediated by family communication.

		Communication time		Perceived communication adequacy	
		β	95% CI	β	95% CI
Family harmony	Indirect effect (with mediator)	0.02	−0.01, 0.05	**0.10**	0.03, 0.18**
	Direct effect (without mediator)	**0.30**	0.01, 0.59*	0.21	−0.08, 0.51
	Total effect	**0.32**	0.02, 0.61*	**0.32**	0.02, 0.61*

Family happiness	Indirect effect (with mediator)	0.02	−0.01, 0.06	**0.12**	0.04, 0.20**
	Direct effect (without mediator)	0.26	−0.05, 0.56	0.16	−0.14, 0.46
	Total effect	0.28	−0.03, 0.59	0.28	−0.03, 0.59

Family health	Indirect effect (with mediator)	0.02	−0.02, 0.06	**0.10**	0.02, 0.17*
	Direct effect (without mediator)	0.07	−0.26, 0.40	−0.01	−0.33, 0.32
	Total effect	0.09	−0.24, 0.42	0.09	−0.24, 0.42

*CI, confidence interval*.

*P < 0.05 are marked in bold*.

*^a^Adjust for age and education*.

*Statistically significant at P < 0.05.

**Statistically significant at P < 0.01.

The improvement in family communication was also found in focus group interviews. The programs provided valuable opportunities for the participants to interact with their family members. They reported that they not only gained an immediate sense of happiness and togetherness during the program activities, family communication also enhanced after that.

(I now) spend more time for communicating. He (the child) is busy with his homework. We don’t have much time to chat. (Now) I spend more time in knowing more about his study. We (the father and the son) can then be happier. (A father, Group 4, 234Z)

Sometimes when I go back home, I chat with him (my son). In the past, I seldom interacted with him. Now, I initiate the conversation…that means we have an improvement in communication. (A mother, Group 2, 272A)

In addition to increased time for communication, the participants were also more open to share the happiness and sadness they encountered in daily life.

Now he (the kids) tells whenever he is happy or sad. That is, he tells me ‘How I feel.’ I can also share (the feeling) with him. When I am upset from work, I also share with him (the kid) too. (A father, Group 6, 302Z)

I communicate more with her (the mother). I tell her the happy and unhappy thins encountered in school. (A son, Group 6, 34C)

The participants reported that their family became more harmonious. There were fewer disputes and the participants knew how to get along with their family members. As the concepts of family 3Hs were interlocking, the happiness level also increased with a more harmonious family. Some participants became more aware of the importance of happiness and health as well.

(I) get along better with the elderly in our family. (Before the programs) I was not sure how the elderly thought. (Now, after the programs) I have an idea of how the elderly and kids think. (The family is) more harmonious. (A father, Group 4, 265A)

After participating in the activity, I think that happiness and health are the most important. (A wife, Group 1, 282A)

(The family is) more harmonious. That means there are fewer arguments. (The family is also) happier. (A father, Group 4, 265A)

## Discussion

Our intervention was effective in improving family communication and family well-being with a small effect size. Perceived communication adequacy substantially mediated the effect of intervention on perceived family 3Hs. The quantitative measures were corroborated by the qualitative assessments which helped evaluate the outcomes more comprehensively.

Previous intervention programs using the social ecological model often contained multiple intensive sessions (27, 28). However, our resident training programs were very brief with only one core session and one booster session (total 5 h). As expected, primary prevention interventions have small effects probably because of the low intensity of the intervention. However, these interventions are most useful when they can be disseminated to large numbers of people. From the public health point of view, brief interventions are expected to attract more participants, and enhance the feasibility and retention rate, as well as reduce the cost of training the interventionists and delivering the programs.

With the involvement of family members in our resident training programs such as workshops and interactive games, our intervention has shown effectiveness on improving family communication, family harmony, and family happiness. Possible reasons may be that these programs provided a platform for the participants to learn and communicate with family members as well as chat and share happy moments. They also learnt to discover and appreciate the strengths of their family members from the program activities.

Moreover, our study also adds to the literature by showing that family communication might be the pathway by which the intervention improved family well-being. The plausible explanation that perceived adequacy of family communication could determine family well-being is that family communication is closely related to the development of intimacy and closeness within families (40, 41), coping with stress and conflict (42, 43), as well as family problem solving and decision making (44, 45). However, communication time did not mediate the effect of intervention on family well-being possibly for several reasons. First, it may be difficult for the participants to recall communication time accurately, introducing random errors into measurement. Second, communication time does not necessarily indicate the quality of communication. Third, Hong Kong people have long working hours, and the small increase in family communication time may not be adequate to improve family well-being.

Our study has several limitations. First, although we chose two well-separated estates, we could not rule out contamination. Second, there were some differences between the intervention and control groups with regard to age and education because individual randomization was not practicable. However, we have adjusted for age and education when assessing the effectiveness of the intervention and the mediation effect of family communication. Third, loss of follow up in the intervention group at 6-week follow-up was about 30%, larger than other studies with similar intensity of intervention in our FAMILY Project (20, 29). Future studies may consider some more appealing incentives to increase the retention rate. Fourth, there could also be some ceiling effects as some participants came with already high score in family well-being before the programs.

There is a Chinese saying that “harmony in family brings success to everything,” reflecting the importance of family harmony in Chinese culture (46). This beneficial effect was substantially mediated by perceived adequacy of family communication. Further research and public education programs focusing on enhancing the quality of family communication are warranted. In addition, health and social service professionals working with families may emphasize the importance of family communication on family well-being to their clients.

Our community-based residents training programs based on the social ecological model improved family communication and perceived family harmony and happiness. Furthermore, perceived adequacy of family communication substantially mediated the effect of the intervention on family well-being, indicating that family communication might be a potential target for further intervention studies to promote family well-being.

## Ethics Statement

Ethical approval was granted by the Institutional Review Board (IRB) of the University of Hong Kong/Hospital Authority Hong Kong West Cluster on November 2010. Written informed consent was obtained from all participants before the start of the programs. For participants younger than 15, written consent was obtained from the next of kin, caretakers, or guardians on their behalf.

## Author Contributions

CS led the development of this manuscript. SC, AW, LK, and TL contributed to the research design. SP and XW helped to collect data. CS analyzed the data. SS made continual input as the draft progressed and approved the final draft for submission. TL and SS critically reviewed the final draft. All authors read and approved the final manuscript.

## Conflict of Interest Statement

The authors declare that the research was conducted in the absence of any commercial or financial relationships that could be construed as a potential conflict of interest.
